# Effectiveness of antiseizure medications therapy in preventing seizures in brain injury patients: A network meta-analysis

**DOI:** 10.3389/fphar.2022.1001363

**Published:** 2022-09-15

**Authors:** Xianhao Huo, Xingguo Xu, Mei Li, Lifei Xiao, Yangyang Wang, Wenchao Li, Chaofan Wang, Tao Sun

**Affiliations:** ^1^ Neurosurgery Department, General Hospital of Ningxia Medical University, Yinchuan, China; ^2^ Ningxia Key Laboratory of Cerebrocranial Disease, Ningxia Medical University, Yinchuan, China; ^3^ Clinical Medical College, Ningxia Medical University, Yinchuan, China; ^4^ Neurosurgery Department, The First Affiliated Hospital of Xinxiang Medical University, Weihui, China

**Keywords:** traumatic brain injury, post-traumatic epilepsy, antiepileptic drugs, PHT, LEV

## Abstract

**Purpose:** To explore the effectiveness of different anti-seizure medications in preventing early and late post-traumatic epilepsy (PTE). The efficacy, treatment-related side-effects, and mortality of the different treatments were compared using a ranking model to identify the optimal treatment.

**Methods:** A comprehensive literature search was performed using Pubmed, Medline, Embase, and Cochrane library databases. All relevant published articles up to 10 March 2022 were evaluated. The quality of the extracted data was assessed using either the Cochrane risk of bias tool or the Newcastle-Ottawa scale. The primary outcome measures were early or late post-traumatic seizures. The secondary outcome measures were mortality, treatment-related adverse effects, length of hospital stay, and length of stay within the intensive care unit (ICU).

**Results:** A total of seven randomized controlled trials and 18 non-randomized controlled trials were included in this network meta-analysis. The trials included six interventions: Phenytoin (PHT)+phenobarbital (PB), levetiracetam (LEV), PHT, PHT-LEV, lacosamide (LCM), and valproate (VPA). All interventions except VPA significantly reduced the rate of early PTE in TBI patients compared with the placebo. Seven studies reported the impact of four treatments (PHT + PB, LEV, PHT, VPA) on late seizures and showed a significant reduction in the incidence of late seizures in patients with TBI compared with placebo. The impact of PHT, LEV, and VPA on mortality was reported in nine studies. PHT had no impact on mortality, but patients treated with both LEV and VPA had higher mortality than those treated with placebo. The treatment-related adverse effects of LEV, PHT, and LCM were reported in five studies. LEV and PHT had higher treatment-related adverse effects incidence than placebo, while LCM had no effect on treatment related-adverse effects.

**Conclusion:** LEV and PHT prevented early and late PTE. PHT also reduced the mortality rate in patients with TBI. Both LEV and PHT had higher treatment-related adverse effects compared with placebo. However, LEV had a slightly lower incidence of treatment-related adverse effects when compared with PHT. Compared with PHT, LEV did not reduce the length of hospital stay but shortened the length of ICU stays. Therefore, based on the findings of this meta-analysis, we speculate that LEV is the best treatment option for TBI patients. However, further high-quality randomized controlled trials are required to confirm these findings.

## 1 Introduction

Traumatic brain injury (TBI) is mainly caused by direct or indirect external forces on the head. More than 50 million people worldwide suffer from TBI each year. Common causes of TBI include car accidents, injuries from falls, and heavy blows to the head (Winkler et al., 2016; Vella et al., 2017). These injuries can result in various disabilities, including neurological deficits, memory loss, and other negative results, making TBI a chronic health condition and a global healthcare burden (Yuan and Wang, 2020).

Post-traumatic epilepsy (PTE) is a recognized complication of TBI. Depending on the location and severity of the bleeding, PTE can occur immediately within 24 h after trauma, early within the first 7 days following trauma, and late after 7 days following trauma (Turnbull et al., 2016; Chartrain et al., 2017). Over the past 30 years, the cumulative incidence of PTE was 2% for mild brain injury, 4% for moderate brain injury, and 15% for severe brain injury (Saletti et al., 2019; Liou et al., 2020). PTE following TBI may further exacerbate the effects of TBI on memory and cognition, damage the cerebrovascular system or blood-brain barrier, and lead to depression or post-traumatic stress disorder (Castriotta et al., 2007; Luo et al., 2014; Muccigrosso et al., 2016; Vos et al., 2018). PTE also makes treating the primary injury more difficult and increases the costs associated with treatment, imposing a serious economic and life burden on the patients’ families and society. Therefore, the prevention of PTE is an important clinical goal in the treatment of TBI.

Prophylactic treatment with anti-seizure medications (ASMs) is increasingly being used to reduce the risk of developing PTE following TBI. The main ASMs used in clinical practice include phenytoin sodium (PHT) (Harris et al., 2020), sodium valproate (VPA) (Ma et al., 2010), phenobarbital (PB) (Servit and Musil, 1981), lamotrigine (LAM) (Kaufman, 2011), levetiracetam (LEV) (Khan et al., 2016), oxcarbazepine (OCBZ) (Degrauw et al., 2018), topiramate (TPM) (Courchia et al., 2018), and carbamazepine (CBZ) (Kirmani et al., 2016). Among these, PHT, OCBZ and CBZ have the common notion of the mechanism, which was reduced high-frequency repetitive discharges of action potentials by enhancing sodium channel inactivation. VPA has multiple mechnisms of action, including GABA potentiation, blocking of T-type calcium channels, and blocking of sodium channels. The main mechanism of PB in preventing seizures is through binding the *γ*-aminobutyric acid (GABA)-A receptor, prolong the opening of the associated chloride channel. The mechanism of LAM is blocked sodium channels, and reduces Ca^2+^-mediated transmitter release. The main mechanism of LEV is binding to the synaptic vesicle protein SV_2_A; inhibits high-voltage-activated Ca^2+^ channels; reverses the negative allosteric effect of GABA/Gly receptor antagonists. TPM has multiple mechanisms of blocking Na^+^ channels, increasing *γ*-aminobutyric acid-mediated inhibition and blocking glutamate-mediated neural excitation, affecting Cl^−^ membrane operation and Ca^2+^ channel blockade. Based on the different mechanisms of the different ASMs and the limitation of the available evidence, the use of ASMs for the prevention of epileptic seizures after TBI is still controversial. A propensity score analysis conducted by Liou et al. (Liou et al., 2020). Revealed that ASMs were ineffective in preventing seizures after TBI, and the benefit of routine prophylactic ASMs treatment in TBI patients needs to be reassessed (Saletti et al., 2019). Studies have shown that VPA was associated with higher mortality in patients with TBI (Temkin et al., 1999), while carbamazepine and PHT were associated with severe adverse effects. As a result, there is a need to identify the optimal ASM therapy to prevent epileptic seizures in TBI patients. ASMs such as LEV and PHT are increasingly being used in clinical practice due to the many favorable features of these drugs. The Brain Trauma Foundation (BTF) guidelines have acknowledged the potential role of PHT and LEV in the management of early PTE but did not provide any clinical recommendations on using these drugs due to insufficient evidence (Carney et al., 2017). As a result, the fourth edition of the BTF guidelines recommends using preventive PHT in the first week following TBI. Still, it does not provide any recommendations for the use of ASMs as a prophylactic treatment for late epileptic seizures (Carney et al., 2017).

In recent years, several studies have been published evaluating the use of ASMs following TBI. However, the current evidence is based on low-quality studies with small sample size. This highlights the need for a meta-analysis to evaluate the current evidence and provide a less biased and more accurate estimation of the clinical problem (Guyatt et al., 1995; Lee, 2018). A network meta-analysis would be ideal in this case since it can be used to compare multiple interventions indirectly by setting a common control group for analysis.

Therefore this study aimed to perform a ranked network meta-analysis to evaluate the effectiveness of ASMs in preventing early or late seizures in TBI patients. In addition, the mortality rate and treatment-related adverse effects of the various therapies were also evaluated.

## 2 Subjects and methods

The systematic review and network meta-analysis were performed according to the checklist of the Preferred Reporting Items for Systematic Reviews and Meta-analyses (PRISMA) extension statement for network meta-analysis.

### 2.1 Inclusion and exclusion criteria

The inclusion and exclusion criteria for this study were based on the PICOS strategy (P: patient/population, I: intervention, C: comparison/control, O: outcome, S: study design). The population criteria of the included literature were patients above the age of 14 years who suffered a TBI(including the presence of subarachnoid hemorrhage, epidural hematoma, subdural hematoma, parenchymal hemorrhage, diffuse axonal injury, and depressed skull fracture confirmed by CT scan), with a time to injury shorter than 24 h. The interventions involved the use of ASMs such as phenytoin, valproate, PB, LAM, LEV, oxcarbazepine, topiramate, and carbamazepine. The outcome indicators were early seizures, late seizures, mortality, and adverse effect. The meta-analysis included randomized controlled trials, prospective cohort, retrospective, or observational studies.

Studies that evaluated the use of ASMs in patients with non-TBI (e.g., cerebral infarction, brain tumor, etc.), spontaneous cerebral hemorrhage, and a history of seizures or trauma were excluded. In addition, studies that included patients treated with ASMs prior to injury or between injury, pregnant or lactating women, and patients with a history of food and drug allergies, severe cardiac, hepatic, or renal dysfunction, chronic alcohol or drug abuse, and severe psychiatric disorders were also excluded. Furthermore, case reports, single-arm studies, literature reviews, letters to the editor, related trials on children, animal experiments, and studies that did not report the outcome indicators were also excluded.

### 2.2 Outcome measures

The primary outcome measures were early or late post-traumatic seizures. The secondary outcome measures were mortality, treatment-related side effects, and length of hospital and intensive care unit (ICU) stay.

### 2.3 Literature search

A comprehensive literature search of studies published up to 10 March 2022, was conducted using Pubmed, Cochrane Library, Embase, and Medline. The keywords used for the search were “brain injury”, “head injury”, “brain hemorrhage”, “parenchymal hemorrhage”, “intracranial hemorrhage”, “subarachnoid hemorrhage”, “epidural hemorrhage”, “anti-epileptic”, “anticonvulsant”, “antiseizure”, “Phenytoin”, “Valproate”, “Phenobarbital”, “Lamotrigine”, “Levetiracetam”, “Oxcarbazepine”, “Topiramate”, and “Carbamazepine”. The references of relevant published systematic reviews were searched manually to identify additional literature. The World Health Organization (WHO) clinical trial registry was also searched manually to identify ongoing and completed unpublished clinical trials evaluating the use of ASMs in TBI patients.

### 2.4 Data screening and quality evaluation

Two professionally trained researchers independently screened all retrieved literature separately. The quality of the included randomized controlled trials was assessed using the Cochrane risk of bias tool. This tool assesses the quality of clinical trials based on six aspects; randomization, allocation concealment, blind application, data completeness, selective reporting, and other biases. The included prospective and retrospective non-randomized controlled trials were evaluated using the Newcastle-Ottawa Scale (NOS). The NOS tool assesses the quality of studies from three aspects: selectivity, comparability, and outcomes. Any disagreements encountered during the screening for relevant articles, quality assessment, and data analysis were resolved through consultations. A third researcher was consulted whenever the two researchers failed to reach an agreement. The quality of the evidence was finally assessed using the Grading of Recommendations Assessment and Development and Evaluation (GRADE) framework.

### 2.5 Data extraction

The author, publication year, country, study type, age, and intervention measures were extracted from each article. In addition, the total number of patients, details of drug treatment, the incidence of PTE, treatment-related adverse effects, and the mortality for each treatment group were also extracted. For studies with missing data, the author of the published research was contacted to obtain additional information.

### 2.6 Statistical analyses

Heterogeneity testing was carried out for all the included articles. The fixed-effect model was used for non-heterogenous studies with a *p* > 0.1 and *I* ^*2*^ < 20%. For all other studies, the random-effects model was adopted. The surface under the cumulative ranking curve (SUCRA) was used to rank the treatment effects. In addition, the node-splitting method was used to conduct a consistency test to determine whether the direct and indirect evidence could be combined. The statistical analyses were performed by Revman Software (Version 5.3; The Cochrane Collaboration) and Stata (version16.0; Corporation, College Station, TX). A 2-tailed *p*-value below 0.05 was considered statistically significant for all statistical tests.

## 3 Results

### 3.1 Literature search results

**FIGURE 1:**
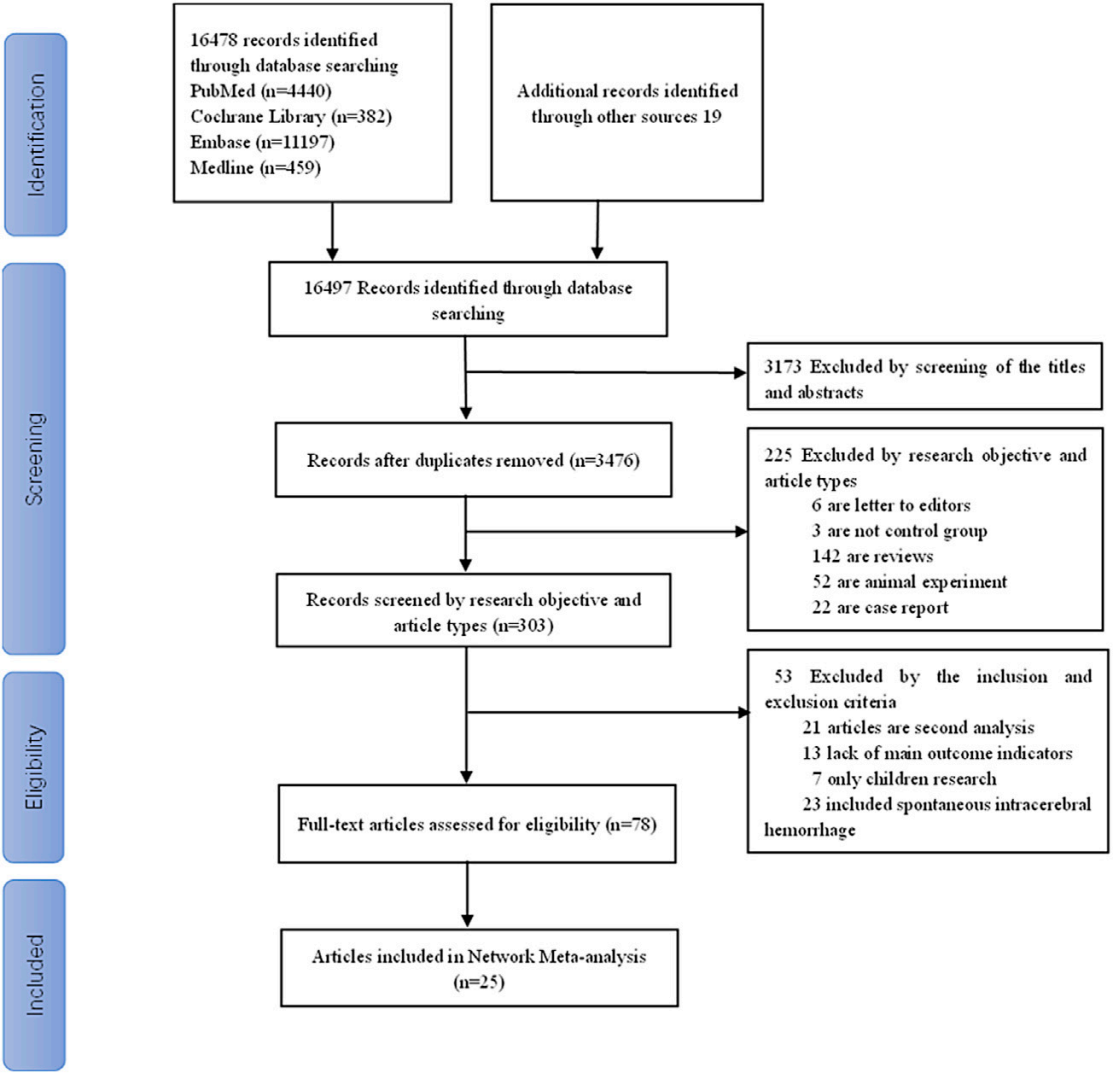
Flow chart of the study selection process.

A total of 16,497 articles were initially retrieved, of which 13021were duplicated and were therefore excluded from the meta-analysis. Another 225 papers were excluded as the research purpose and/or the literature type were not in line with the aims of this meta-analysis. An additional 53 articles were excluded as these did not meet the eligibility criteria of this meta-analysis. Finally, 25 articles with a total sample size of 6,466 cases were included in the network meta-analysis, including seven randomized controlled trials (Young et al., 1983a; Young et al., 1983b; Temkin et al., 1990; Temkin et al., 1999; Szaflarski et al., 2010; Khan et al., 2016; Younus et al., 2018), four prospective studies (Jones et al., 2008; Inaba et al., 2013; Gabriel and Rowe, 2014; Khor et al., 2018), 13 retrospective studies (Wohns and Wyler, 1979; Servit and Musil, 1981; Ma et al., 2010; Debenham et al., 2011; Caballero et al., 2013; Kruer et al., 2013; Bhullar et al., 2014; Javed et al., 2016; Zangbar et al., 2016; Hazama et al., 2018; Kwon et al., 2019; Harris et al., 2020; Nguyen et al., 2021), and one non-randomized trial (Klein et al., 2012) as shown in Figure 1. The characteristics of the study design, interventions, and sample size for each treatment group are summarized in (Tables 1, 2). The studies evaluated six interventions, including PHT + PB, LEV, PHT, PHT-LEV, LCM, and valproate.

**TABLE 1:** Baseline characteristics of involved patients.

Study	Country	Publication year	Age(y) mean	Male (%)	Numbers	End points	Follow-up time
Temkin NR^[39]^	Seattle	1990	34 ± 18	78%	208/196	Early and late seizures, adverse effects	2 years
			34 ± 17	75%			
Wohns RN^[24]^	Washington	1979	29 ± 19	75.8%	50/12	Late seizures	6 years
Servít Z^[14]^	Czechoslovakia	1981	30.6	76.6%	143/24	Late seizures	8–13 years
Young B^[25]^	Kentucky	1983	24.4 ± 1.29	80.9%	136/108	Early seizures	NA
			25.8 ± 1.47	84.3%			
Young B^[26]^	Kentucky	1983	24.1 ± 1.47	81.2%	85/74	Late seizures	18 months
			26.3 ± 2.03	75.0%			
Temkin NR^[20]^	Seattle	1999	40 ± 19	84%	120/127/132	Early seizures, late seizures, mortality, and adverse effect	2 years
			36 ± 16	77%			
Jones KE^[40]^	Pennsylvania	2008	≥25	73%	15/12	Early seizures	6 months
			≥25	75%			
Debenham S^[28]^	United States	2011	55.0 ± 22.3	NA	653/355	Early seizures, adverse effects	2 years
			46.9 ± 21.3	NA			
Szaflarski JP^[27]^	United States	2010	35	72.2%	34/18	Seizures, mortality, adverse effects	6 months
			34	76.5%			
Ma CY^[13]^	China	2010	≥18	76.7%	35/124	Early seizures	NA
Inaba K^[43]^	California	2012	51.7 ± 21.3	73.9%	406/407	Seizures, adverse effect, mortality, complications	33 months
			53.6 ± 22.5	68.8%			
Klein P^[41]^	United States	2012	6–70	75.8%	66/60	Seizures, adverse effect	2 years
			6–70	81.7%			
Caballero GC^[37]^	Texas	2013	50	75.6%	18/72	Seizures, LOS of ICU	NA
			57	77.8%			
Kruer RM^[29]^	United States	2013	43.1	85.4%	20/89	Early seizures, mortality	NA
			34.1	95%			
Bhullar IS^[30]^	United States	2013	41 ± 18	84%	50/43	Early seizures, mortality, LOS of ICU and hospital	NA
			36 ± 16	65%			
Gabriel WM^[31]^	United States	2014	48.8 ± 24.2	60%	5/14	Early and late seizures, adverse effect	6 months
			46.8 ± 16.9	71.4%			
Javed G^[42]^	Pakistan	2016	31.16 ± 17.39	NA	50/50	Early seizures	NA
			34.96 ± 18.26				
Zangbar B^[32]^	United States	2016	47 ± 23	72.6%	208/208	Seizures	3 years
			45 ± 25	64.4%			
Khan SA^[16]^	Pakistan	2016	24.15 ± 9.56	39.0%	77/77	Early seizures	1 week
				35.7%			
Khor D^[33]^	China/United States	2018	46 (27.5–64)	74.6%	272/250	Early seizures	1 week
			44 (30–54)	77.2%			
Hazama A^[36]^	United States	2018	32 ± 16	76%	227/176	Early seizures	6 months
			33 ± 15	67%			
Younus SM^[35]^	Pakistan	2018	29.48 ± 16.24	83.6%	70/70	Early seizures	6 months
Kwon SJ^[34]^	Phoenix	2019	58 ± 22	71%	365/116	Early seizures, adverse effect	NA
			50 ± 21	66%			
Harris L^[12]^	United Kingdom	2020	53.7 (15–95)	74.0%	77/23	Early seizures, LOS of hospital, adverse effect	NA
			63.7 (22–89)	78.2%			
Nguyen JV^[38]^	United States	2021	54.6 (35.6–71.4)	66.7%	105/95	Early and late seizures, LOS of hospital and ICU, mortality	NA
			53.2 (31–68.7)	76.8%			

PHT: phenytoin; LEV, levetiracetam; LOS, length of stay; y, year; NA, not report.

**TABLE 2:** Overview of the included literature.

Study	Publication year	Study design	Study type	Intervention	Treatment details
				Control	
Temkin NR	1990	Randomized, double-blind study	Monocenter	PHT	PHT or placebo was administered intravenously within 24 h of injury, the initial dose of medication was 20 mg/kg, the doses given of intravenously or orally ranged from 200 to 1200mg, and the doses of suspension given through a nasogastric tube were as high as 2600 mg
				Placebo	
Wohns RN	1979	Retrospective study	Monocenter	PHT	PHT was given with the most common dose of 400 mg/d
				Placebo	
Servít Z	1981	Retrospective study	Monocenter	PHT + phenobarbital	The treatment group: PHT (160–240 mg/d) and phenobarbital (30–60 mg/d) for at least 2 years after head trauma. The control group was given placebo
				Placebo	
Young B	1983	Randomized, double-blind, placebo- controlled study	Monocenter	PHT placebo	The initial PHT dose of 11 mg/kg was intravenously, and then 13 mg/kg of PHT was administered intramuscularly in divided doses at multiple sites. The control group was given placebo
Young B	1983	Randomized, double-blind, placebo-controlled study	Monocenter	PHT placebo	The initial PHT dose of 11 mg/kg was intravenously, and then 13 mg/kg of PHT was administered intramuscularly in divided doses at multiple sites. The control group was given placebo
Temkin NR	1999	Randomized, double-blind, placebo-controlled study	Monocenter	Valproate	Phenytoin was intravenously administered loading dose of 20 mg/kg, with maintenance dosing beginning at 5 mg/kg/d in two divided doses. The valproate was intravenously administered loading dose of 20 mg/kg, with initial maintenance dosing of 15 mg/kg/d in four divided doses
				PHT	
Jones KE	2008	Prospective cohort study	Monocenter	LEV	LEV was intravenously administered 500 mg every 12 h for the first 7 days. The PHT was intravenously administered for 7 days
				PHT	
Debenham S	2011	retrospective study	Monocenter	PHT placebo	PHT was intravenously administered with a loading dose of 17 mg/kg over 30–60 min, followed by a maintenance dose of 100 mg given three times daily, either intravenously or orally for a total of 7 days
Szaflarski JP	2010	Randomized single blind, placebo-contrelled study	Monocenter	LEV	LEV was intravenously administered with a loading dose of 20 mg/kg, rounded to the nearest 250 mg over 60 min then started on maintenance dose (1000mg, IV every 12 h given over 15 min). PHT was intravenously administered with a loading dose of fos-PHT 20 mg/kg, maximum of 2000 mg, given over 60 min and was then started on a PHT maintenance dose (5 mg/kg/d, rounded to nearest 100 mg dose, IV every 12 h given over 15 min)
				PHT	
Ma CY	2010	Retrospective study	Monocenter	Valproate placebo	Valproate was intravenously administered with a loading dose of 10–15 mg/kg/d. The control group was not received treatment
Inaba K	2012	Prospectively study	Multicenter	LEV	LEV was intravenously administered with 1000 mg, every 12 h given over 15 min. PHE was intravenously administered with a loading dose of 20 mg/kg given at 50 mg per min and then started on a PHT maintenance dose (5 mg/kg/d, rounded to the nearest 100 mg, every 8 h given over 15 min)
				PHT	
Klein P	2012	Non-Randomized phase Ⅱ study	Monocenter	LEV placebo	LEV was intravenously administered with 55 mg/kg/d in 2 divided doses at 8 a.m. and 8 p.m. for 30 days
Caballero GC	2013	Retrospective study	Monocenter	LEV	LEV was intravenously administered with a loading dose of 1000mg, and maintenance dose of 500 mg every 12 h. PHT was intravenously administered with a loading dose of 10–17 mg/kg, and maintenance dose of 3–4.5 mg/kg/d
				PHT	
Kruer RM	2013	Retrospective observational study	Monocenter	LEV	LEV and PHT was intravenously administered for 7 days
				PHT	
Bhullar IS	2013	Retrospective study	Monocenter	PHT placebo	PHT was intravenously administered for 7 days
Gabriel WM	2014	Prospective cohort study	Monocenter	LEV	LEV was intravenously administered with a 500 mg twice daily. PHT was intravenously administered with a loading dose (14.6 ± 1.93 mg/kg) that was followed by a maintenance dose (4.4 ± 0.5 mg/kg/d)
				PHT	
Javed G	2016	Retrospective cohort study	Monocenter	PHT-LEV	PHT was intravenously administered for 2 days and on the third day was switched to enteral LEV at the dose of 35 mg/kg per dose. The control group was given PHT
				PHT	
Zangbar B	2016	Retrospective study	Monocenter	LEV	LEV was intravenously administered for 7 days
				Placebo	
Khan SA	2016	Randomized controlled trial	Monocenter	LEV	LEV was intravenously administered with 20 mg/kg over 60 min followed by maintenance dose of 10–20 mg/kg/d in two divided doses. PHT was intravenously administered with a loading dose of 20 mg/kg over 60 min followed by maintenance dose of 5 mg/kg/d in two divided doses
				PHT	
Khor D	2018	Prospective observational study	Multicenter	LEV placebo	LEV was intravenously administered with a dose of 500 mg every 12 h for 7 days
Hazama A	2018	Retrospective cohort study	Monocenter	LEV placebo	LEV was intravenously administered with a dose of 500 mg every 12 h for 7 days
Younus SM	2018	Randomized controlled trial	Monocenter	LEV	LEV was intravenously administered with a dose of 1000 mg, followed by a dose of 500–1000 mg twice daily. PHT was intravenously administered with a dose of 15–20 mg/kg, followed by a dose of 4–8 mg/kg divided into three doses per day
				PHT	
Kwon SJ	2019	Retrospective cohort study	Monocenter	Lacosamide	Lacosamide was given either 50 mg twice daily or 200 mg once, followed by 100 mg twice daily for 7 days. PHT was given 15–20 mg/kg followed by 300–400 mg daily for 7 days
				PHT	
Harris L	2020	Retrospective observational study	Monocenter	LEV	PHT was given 300 mg once daily and LEV was given 500 mg twice a day
				PHT	
Nguyen JV	2021	Retrospective cohort study	Monocenter	LEV	LEV and PHT was intravenously administered for 7 days
				PHT	

PHT: phenytoin; LEV: levetiracetam; LOS, length of stay; y, year; NA, not report.

### 3.2 Quality evaluation

According to the Cochrane risk of bias tool, the seven randomized controlled trials included in this meta-analysis used correct random assignment methods, had complete outcome data information and were not selectively reported. The study by Temkin et al. (1990) was not blinded at trial implementation and outcome assessment, while the study by Szaflarski et al. (2010) was not blinded at outcome assessment. We could not determine whether the blinding method in the studies by Khan et al. (2016) and Younus et al. (2018) was correctly implemented during the intervention and evaluation of the outcome measures. Since it was not possible to determine the level of bias in the included randomized controlled studies, the quality of the studies was classified as moderate (Supplementary Figure S1). The total NOS score of the included 18 non-randomized trials was above 5, indicating high quality within these studies (Supplementary Table S1).

### 3.3 Traditional meta-analysis and GRADE

Twenty-two studies reported the rate of early seizures in patients with TBI after treatment with different ASMs. The results of the subgroup analysis are available in Supplementary Figure S2A. Since the subgroups had significant heterogeneity (*I* ^*2*^ > 20%, *p* < 0.1), the random-effects model was adopted. Patients treated with PHT had a significantly lower rate of early seizures when compared with those treated with placebo (*p* = 0.007). However, the rate of early seizures did not differ significantly between PHT and other ASMs, including LEV, VPA, PHT-LEV, and LCM (All *p* > 0.05). In addition, VPA and LEV had no significant impact on the rate of early seizures compared with the placebo (*p* = 0.31, 0.54, respectively).

Seven studies reported the incidence of late seizures in patients with TBI after treatment with different ASMs. These were analyzed in subgroups as shown in Supplementary Figure S2B. Since the heterogeneity between subgroups (*I* ^*2*^ > 20%, *p* < 0.1) was significant, the random-effects model was adopted. PHT + PB significantly reduced the rate of late seizures compared to a placebo (*p* < 0.05). However, the incidence of late seizures following PHT treatment did not differ significantly from that of LEV, VPA, and a placebo (all *p* > 0.05).

Nine studies reported mortality (the cause is TBI) in patients with TBI after treatment with different ASMs. The results of the subgroup analysis are summarized in Supplementary Figure S2C. Since there is no significant difference in heterogeneity between subgroups (*I* ^*2*^ < 20%, *p* > 0.1), the fixed-effect model was adopted. PHT had no significant impact on mortality compared with the placebo, VPA, and LEV (all *p* > 0.05). The mortality between patients treated with LEV and the placebo did not vary significantly (*p* = 0.56).

The adverse effects of these drugs mainly were fever, cardiovascular, hematologic and dermatologic abnormalities (like bradycardia, myocardial infarction, atrial fibrillation, neutropenia, thrombocytopenia, rash, skin itchiness, etc), abnormal liver and kidney function (like elevated liver enzymes, acute kidney injury, diabetes insipidus, etc), and so on. In this network meta-analysis, five studies reported treatment-related adverse effects in TBI patients after treatment with different ASMs. Because of the details of treatment-related adverse effects reported by these studies were not completely consistent, this study only analyzed the overall incidence of treatment-related adverse effects. The results of the subgroup analysis of these studies are summarized in Supplementary Figure S2D. Since the subgroups had significant heterogeneity (*I* ^*2*^ > 20%, *p* < 0.1), the random-effects model was adopted. PTH did not increase the incidence of treatment-related adverse effects compared with placebo and LEV (*p* = 0.23 and *p* = 0.24), but LCM significantly reduced the incidence of treatment-related adverse effects compared with the PHT (*p* < 0.05).

The length of hospital stay was reported in eight studies, while the length of ICU stay was reported in five studies. The results of the subgroup analysis are summarized in Supplementary Figures S3A, 3B). Since the subgroups had significant heterogeneity (*I* ^*2*^ > 20%, *p* < 0.1), the random-effects model was adopted. LEV did not reduce the length of hospital stay (*p* = 0.37) but significantly reduced the length of ICU stay (*p* = 0.04) compared to PHT. However, PHT significantly prolonged the length of hospital stay compared to the placebo (*p* < 0.05).

The credibility of the evidence for early seizures, late seizures, mortality, and the treatment-related adverse effects was very low in the non-randomized controlled trials due to the use of an observational study design and the indirectness of several comparisons (Tables 3). Therefore further high-quality research is needed to provide more evidence.

**TABLE 3:** (Panel A) GRADE assessment of the quality of evidence based on the RCT. (Panel B) GRADE assessment of the quality of evidence based on the observational study.

Panel A	Certainty assessment						Summary of findings			Quality of evidence
Outcomes	Study design	Risk of bias	Inconsistency	Indirectness	Imprecision	Publication bias	Study event rates			
							With Experimental	With control	Relative risk (95% CI)	
Early seizures	Randomized trials	No serious limitations	No serious limitations	Serious limitations due to indirectness	No Serious limitations	No serious limitations	29/636 (4.6%)	51/493 (10.3%)	0.53 (0.22, 1.28)	⊕⊕⊕○
										Moderate
Late seizures	Randomized trials	No serious limitations	No serious limitations	Serious limitations due to indirectness	No Serious limitations	No serious limitations	134/540 (24.8%)	117/402 (29.1%)	0.97 (0.80, 1.17)	⊕⊕⊕○
										Moderate
death	Randomized trials	No serious limitations	No serious limitations	Serious limitations due to indirectness	No serious limitations	No serious limitations	88/715 (12.3%)	53/454 (11.7%)	1.03 (0.75, 1.43)	⊕⊕⊕○
										Moderate

Panel B	Certainty assessment						Summary of findings			Quality of evidence
Outcomes	Study design	Risk of bias	Inconsistency	Indirectness	Imprecision	Publication bias	Study event rates			
							With Experimental	With control	Relative risk (95% CI)	
Early seizures	Observational studies	No serious limitations	No serious limitations	Serious limitations due to indirectness	No Serious limitations	No serious limitations	81/2,574 (3.1%)	83/2,111 (3.9%)	0.81 (0.60, 1.10)	⊕○○○
										Very low
Late seizures	Observational studies	No serious limitations	No serious limitations	Serious limitations due to indirectness	No Serious limitations	No serious limitations	10/303 (3.3%)	21/165 (12.7%)	0.20 (0.10, 0.38)	⊕○○○
										Very low
Adverse effect	Observational studies	No serious limitations	No serious limitations	Serious limitations due to indirectness	No Serious limitations	No serious limitations	40/1429 (2.8%)	43/892 (4.8%)	0.85 (0.56, 1.27)	⊕○○○
										Very low
Death	Observational studies	No serious limitations	No serious limitations	Serious limitations due to indirectness	No Serious limitations	No serious limitations	43/647 (6.6%)	62/694 (8.9%)	1.18 (0.78, 1.76)	⊕○○○

### 3.4 Network meta-analysis

#### 3.4.1 Network meta-analysis diagram

**FIGURE 2:**
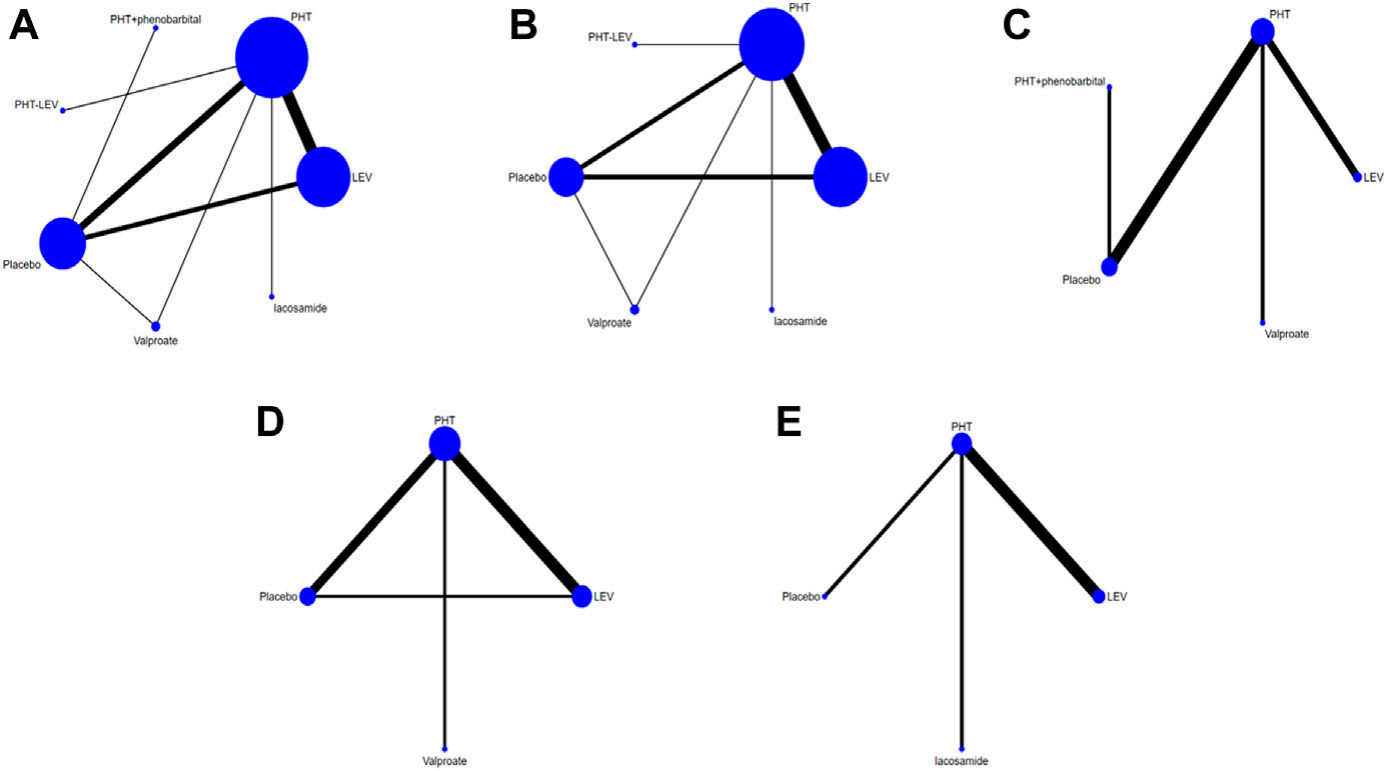
Network Chart; **(A)** The network chart of total seizures, **(B)**. The network chart of early seizures, **(C)**. The network chart of late seizures, **(D)**. The network chart of death, **(E)**. The network chart of the adverse effect.

Figure 2 illustrates a diagram of the network meta-analysis for the different interventions. A direct line between two interventions indicates evidence of direct comparison, while no line indicates no evidence of direct comparison. The size of the dots represents the different sample sizes, and the thickness of the lines represents the number of studies. The PHT, LEV, and placebo treatment groups had the largest sample sizes and the largest number of direct or indirect entries in the comparative trials.

#### 3.4.2 Inconsistency test

Since both direct and indirect evidence was present in this study, consistency testing was performed before integrating the results. No evidence of inconsistency was found in this network model as the differences were no statistically significant (*p* > 0.05). Therefore the included direct and indirect evidence was combined.

#### 3.4.3 Network meta-analysis sequence diagram

**FIGURE 3:**
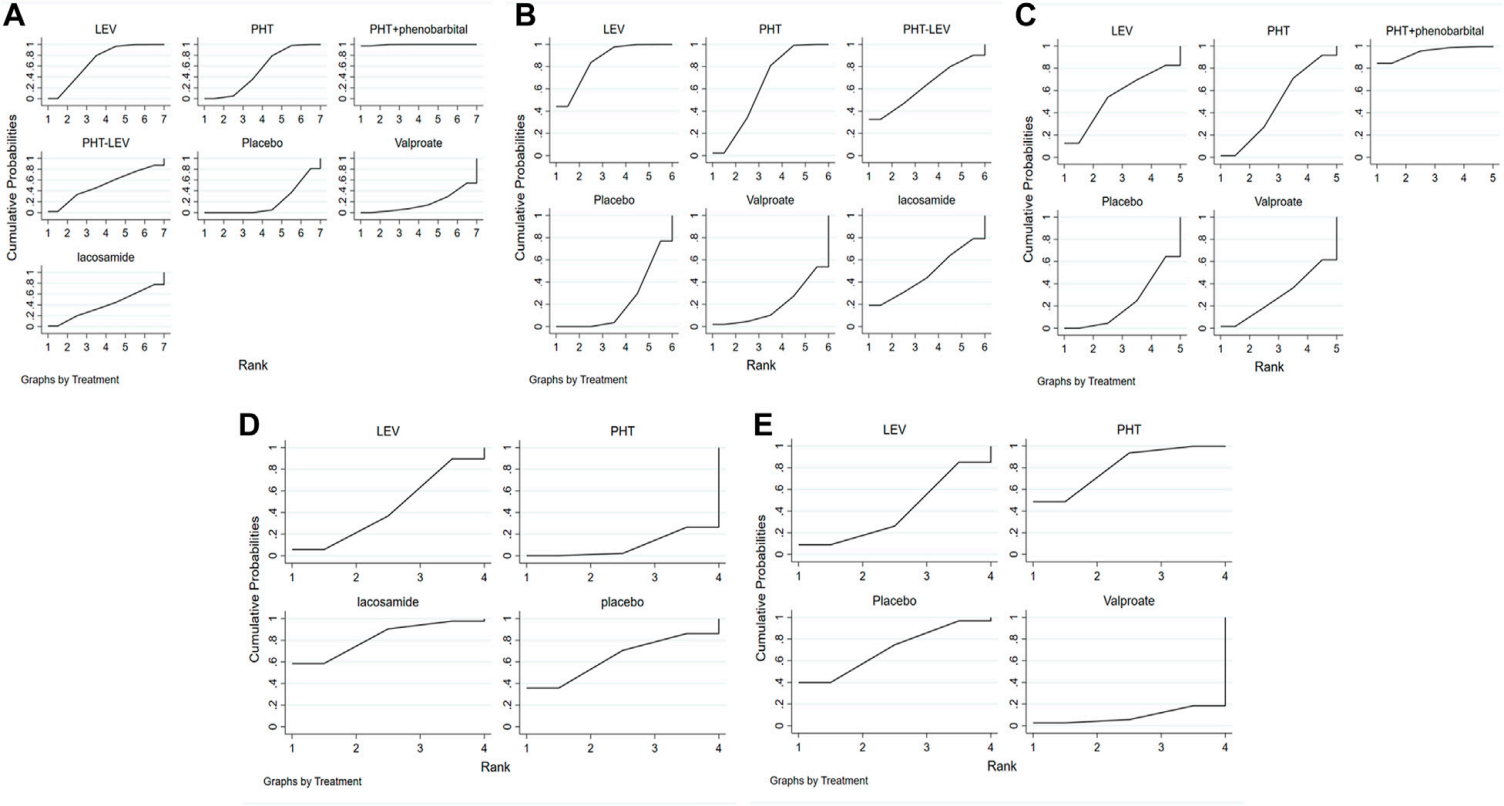
The Rank Chart; **(A)** The rank chart of total seizures, **(B)**. The rank chart of early seizures, **(C)**. The rank chart of late seizures, **(D)**. The rank chart of the adverse effect, **(E)**. The rank chart of death.

Figure 3A shows the network meta-analysis sequence diagram for PTE occurrence after treatment with different ASMs. In this figure the larger area under the curve, this ASM has a lower incidence of PTE. The PTE reduction efficacy of six different ASMs, including PHT + PB, LEV, PHT, PHT-LEV, LCM, and VPA, were compared with a placebo. All treatments except VPA significantly reduced the incidence of PTE in TBI patients. PHT + PB had the highest impact in reducing PTE, followed by LEV, PHT, PHT-LEV, LCM, placebo, and VPA.

The occurrence of early seizures following treatment with five ASMs (LEV, PHT, PHT-LEV, LCM, VPA) was evaluated in 22 studies. Figure 3B shows the network meta-analysis sequence diagram for the rate of early seizures after treatment with different ASMs. In this figure the larger area under the curve, this ASM has a lower rate of early seizures. All treatments except for VPA significantly reduced the rate of early seizures compared to placebo in patients with TBI. LEV had the highest efficacy, followed by PHT, PHT-LEV, LCM, placebo, and VPA. Late seizures were reported in seven studies. Figure 3C shows the network meta-analysis sequence diagram for the rate of late seizures after treatment with different ASMs. In this figure the larger area under the curve, this ASM has a lower rate of late seizures. These studies evaluated four ASMs, including PHT + PB, LEV, PHT, and VPA. All the four regimens significantly reduced the rate of late seizures in patients with TBI compared with placebo. PHT + PB had the highest efficacy in reducing the onset of late seizures, followed by LEV, PHT, VPA.

The overall treatment-related adverse effects were reported in five studies for three different treatment regimes (PHT, LEV, and LCM). This analysis was used to compare the treatment-related adverse effects between different regimes. Figure 3D shows that the larger area under the curve, this ASM has a lower rate of treatment-related adverse effects. All treatment regimes except LCM had significantly higher treatment-related adverse effects when compared with a placebo. PHT had the highest rate of treatment-related adverse effects, followed by LEV, placebo, and LCM. Patient mortality for three treatment regimes (PHT, LEV, and VPA) was reported in nine studies. Figure 3E shows the network meta-analysis sequence diagram for the rate of mortality after treatment with different ASMs. In this figure the larger area under the curve, this ASM has a lower rate of mortality. Therefore, this figure showed that the mortality in patients treated with LEV and VPA was significantly higher than that in patients treated with the placebo, but the mortality in patients treated with PHT was lower that in patients treated with the placebo.

## 4 Discussion

The treatment of PTE in patients with TBI is still controversial. PHT was the historical gold standard for the prevention of PTE, as revealed in a landmark randomized, double-blind, placebo-controlled trial conducted by Kruer et al. (2013). However, although PHT reduced the incidence of early seizures in TBI patients compared with the placebo, it did not reduce the incidence of late seizures or mortality. Conversely, a retrospective study conducted in 2013 by Bhullar et al. (2014) showed that PHT treatment is not effective in reducing early PTE.

For more than a decade, experts in neuroscience have explored the efficacy and safety of various ASMs in the prevention of PTE after TBI. However, the reported efficacy of these treatments in preventing PTE is still inconclusive. As a result, the current treatment for PTE is based on the recommendations of BTF guidelines, clinical experience, and the clinician’s subjective opinions. As a result, there is a need to identify the optimal ASMs treatment for TBI patients.

In this study, we for the first time conducted a comprehensive search of clinical studies related to all drug regimens for preventing seizures after TBI and used the principle of indirect comparison of the Network meta-analysis. By establishing a common control between all the ASMs, to compare the efficacy and safety of different ASMs.

In the beginning, we performed a traditional meta-analysis to compare the onset of early and late seizures and mortality in subgroups of patients treated with different ASMs. Consistent with the trial of Temkin et al. (1999), our findings showed that PHT could reduce the incidence of early seizures but not the incidence of late seizures and mortality in patients with TBI compared with placebo. Therefore, we hypothesized that PHT might be an effective ASM for the prevention of early PTE. Our analysis found no significant differences between PHT and LEV in preventing early and late seizures, treatment-related adverse effects, and mortality in patients with TBI. In the conventional meta-analysis by Zafar et al. (2012), PHT and LEV were found to be equally effective in preventing early and late seizures. However, the studies in the meta-analysis (Zafar et al., 2012) also included patients with brain injury caused by surgery, tumors, and spontaneous brain hemorrhage. Furthermore, Zafar et al. (2012) did not analyze the adverse effects and mortality associated with the two treatments. In a systematic evaluation published in 2016 (Xu et al., 2016), LEV and PHT were also found to be equally effective in preventing seizures in patients with brain injury. Nevertheless, in this study, LEV had a better safety profile than PHT. However, it is important to note that Xu et al. (2016). Included patients with brain injury caused by a spontaneous brain hemorrhage, while our study only included patients with brain injury caused by trauma. Therefore, we believe that the efficacy and safety of LEV and PHT in preventing seizures in patients with TBI still need to be further explored. In addition, we also found that VPA, PHT + LEV, and LCM did not reduce early seizures in patients with TBI compared with PHT. Conversely, VPA and PHT + LEV did not reduce late seizures in patients with TBI, but PHT combined with PB reduced late seizures in patients with TBI. The reliability of these conclusions requires further investigation as these regimens involved only one study with a small sample size and insufficient strength of evidence.

Subsequently, based on the available evidence and the results of the traditional meta-analysis, a network meta-analysis was performed to compare the direct and indirect outcomes of the six treatment regimens (PHT + PB, LEV, PHT, PHT-LEV, LCM, and VPA). This network meta-analysis included 25 studies with a total sample size of 6,466. The SUCRA ranking was used to compare the treatment efficacy, adverse effects, and mortality of all six regimens.

Twenty-two studies evaluated the impact of five treatment regimens, namely LEV, PHT, PHT-LEV, LCM, and VPA, in reducing the incidence of early PTE. All treatment regimes except VPA could significantly reduce the incidence of early PTE compared with placebo. In terms of late seizures, seven studies revealed that PHT + PB, LEV, PHT, and VPA could reduce the late PTE rate in TBI patients compared with placebo.

However, the current network meta-analysis included only one study on PHT combined with PB. The study had a very small sample size, so the role of this regimen in reducing the incidence of seizures after TBI still needs to be further explored.

In the nine studies evaluating patient mortality, LEV and VPA had higher patient mortality than placebo. However, there was no significant difference in the mortality between PHT and the placebo. In the ranking analysis for the five studies that evaluated treatment-related adverse effects, both LEV and PHT had higher treatment-related adverse effects than the placebo. Although PHT and LEV did not reduce the length of hospital stay, they significantly reduced the length of ICU stay.

## 5 Limitations

This network meta-analysis has some limitations that have to be acknowledged. Studies evaluating PHT and LEV were based on very large-scale, high-quality randomized controlled trials. However, studies evaluating the remaining four treatment options involved a small number of studies. Therefore, the relevant ranking of these four treatment options is unreliable and still requires further pooled analysis. This network meta-analysis contains 18 non-randomized controlled trials with a low GRADE rating which may reduce the strength of evidence for the conclusions of this study. The number of studies reporting the incidence of late seizures, mortality, and treatment-related adverse effects was small and mostly involved an observational research design with a small sample size. Therefore the conclusions in this part of the study are less convincing and need to be revalidated by high-quality, large-sample randomized controlled trials. In addition, the overall incidence of treatment-related adverse effects was not explored in most of the six treatment regimens. As a result, we could not perform a clusterank analysis to evaluate the effectiveness and safety of the different treatments and the impact of adverse effects on treatment efficacy.

## 6 Conclusion

LEV and PHT prevented early and late PTE in patients with TBI. PHT also reduced the mortality rate in patients with TBI. Both LEV and PHT had higher treatment-related adverse effects compared with placebo. However, LEV had a slightly lower incidence of treatment-related adverse effects when compared with PHT. Compared with PHT, LEV did not reduce the length of hospital stay but shortened the length of ICU stays. Therefore, based on the findings of this meta-analysis, we speculate that LEV is the best treatment option for TBI patients. However, further high-quality randomized controlled trials are required to confirm these findings.

## Data Availability

The original contributions presented in the study are included in the article/Supplementary Material, further inquiries can be directed to the corresponding author.

## Abbreviations

PHT, phenytoin; PB, phenobarbital; LEV, levetiracetam; LCM, lacosamide; VPA, valproate; LAM, lamotrigine; OCBZ, oxcarbazepine; CBZ, carbamazepine; TPM, topiramate; TBI, traumatic brain injury; PTE, post-traumatic epilepsy; AEDs, antiepileptic drugs; ICU, intensive care unit; BTF, Brain Trauma Foundation; PRISMA, Preferred Reporting Items for Systematic Reviews and Meta-analyses; WHO, World Health Organization; NOS, Newcastle-Ottawa Scale; GRADE, Grading of Recommendations Assessment and Development and Evaluation; SUCRA, surface under the cumulative ranking curve.
