# Supplementary material for: Effectiveness of antiseizure medications therapy in preventing seizures in brain injury patients: A network meta-analysis
Source: Front Pharmacol. 2022 Sep 15;13:1001363. doi: 10.3389/fphar.2022.1001363 (PMC9521202; doi:10.3389/fphar.2022.1001363)
Supplement: Supplementary file 1 [file DataSheet1.docx]

Supplementary Material

# Supplementary Figures


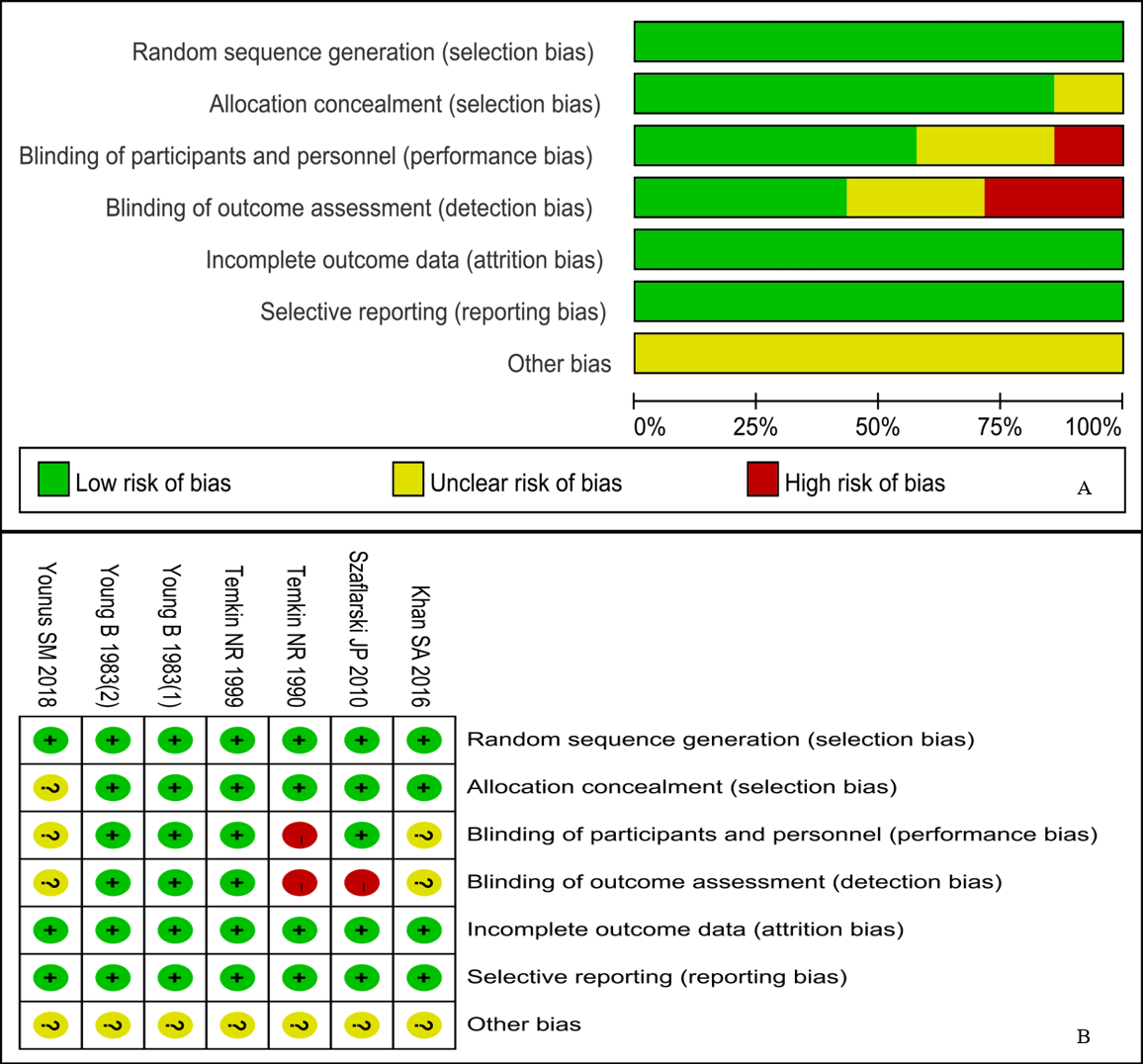


**Supplementary Figure 1**. Quality assessment of identified RCTs


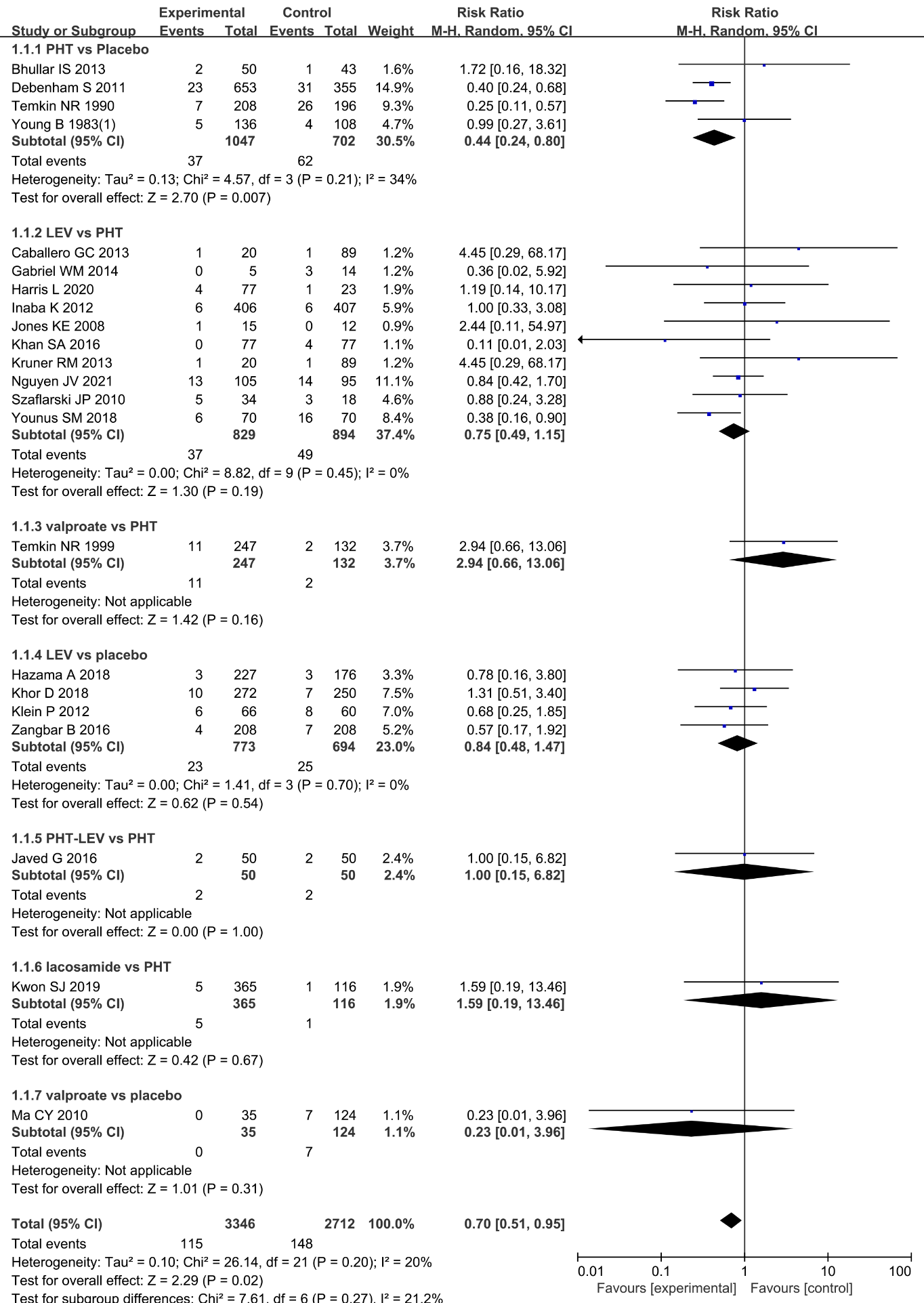


**Supplementary Figure 2a.** The subgroup analysis based on the early seizures


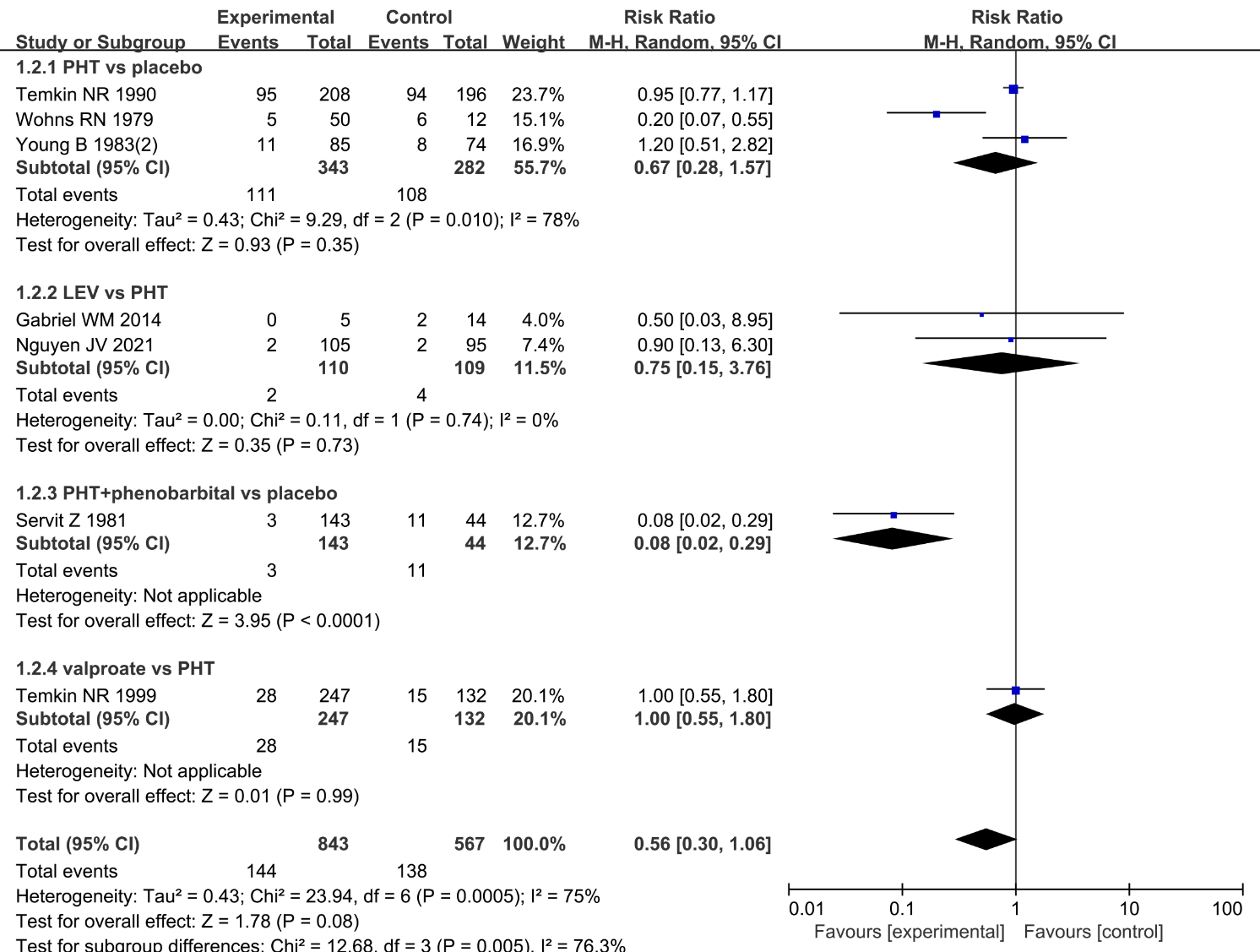


**Supplementary Figure 2b.** The subgroup analysis based on the late seizures


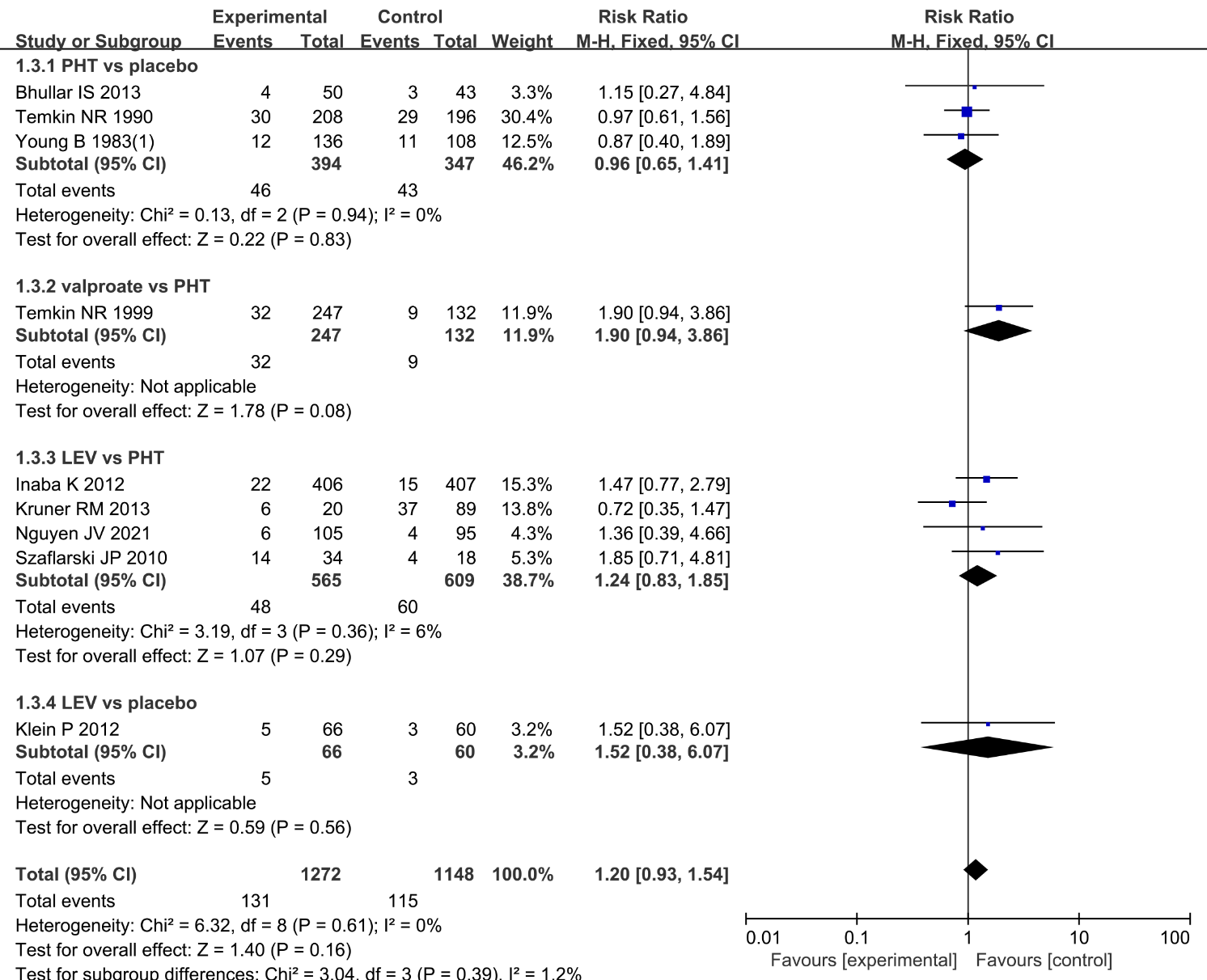


**Supplementary Figure 2c.** The subgroup analysis based on the death


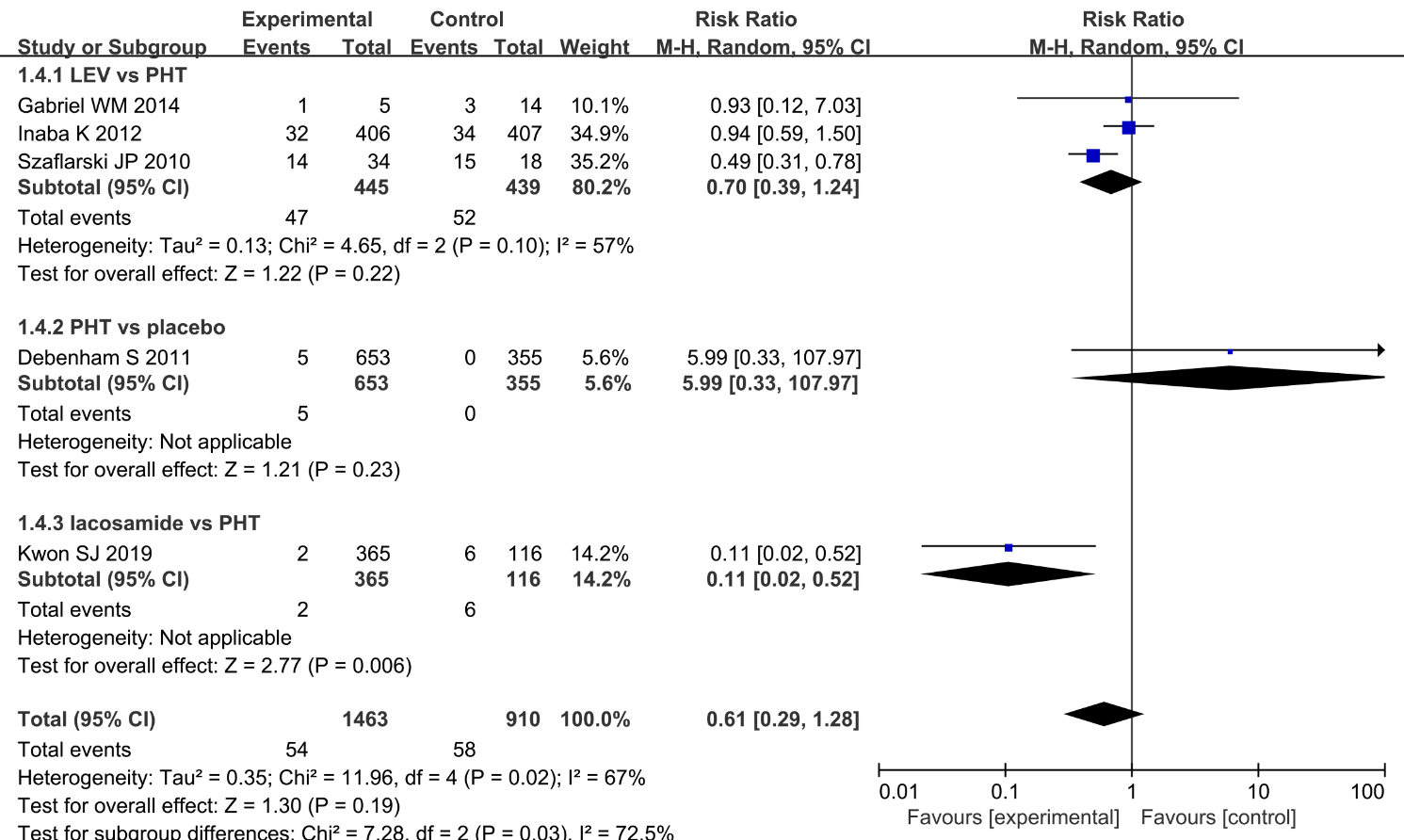


**Supplementary Figure 2d.** The subgroup analysis based on the treatment-related adverse effects


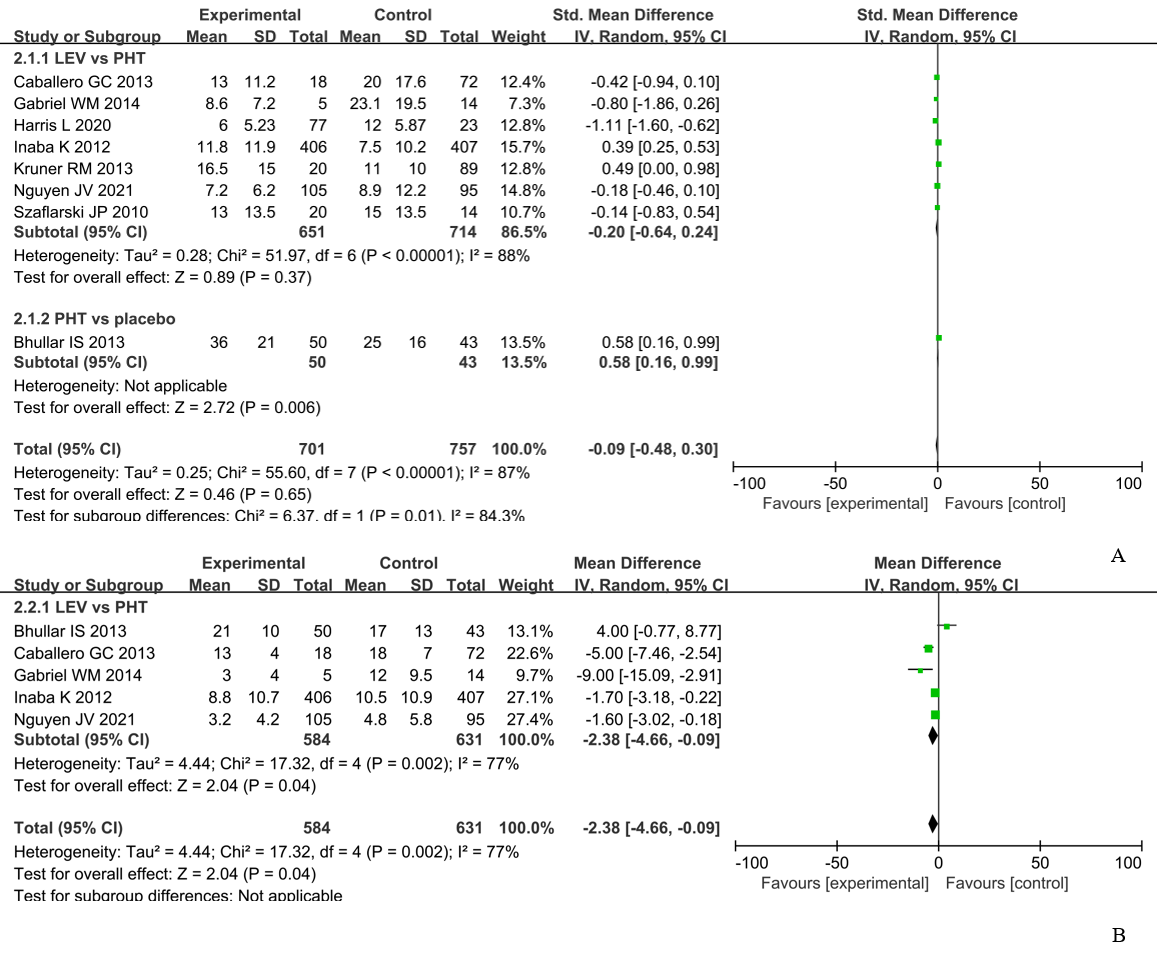


**supplementary Figure 3.** The subgroup analysis based on the LOS of hospital and ICU; A: The subgroup analysis based on the LOS of hospital, B: The subroup analysis based on the LOS of ICU.

# Supplementary Tables

Supplement Table 1. Quality assessment of the non-randomized, controlled trials

| Study | Selection | Comparability | Outcome | Total Score |
| --- | --- | --- | --- | --- |
| Wohns RN | 4 | 2 | 2 | 8 |
| Servít Z | 4 | 2 | 3 | 9 |
| Jones KE | 4 | 2 | 3 | 9 |
| Debenham S | 4 | 2 | 2 | 8 |
| Ma CY | 4 | 2 | 2 | 8 |
| Inaba K | 3 | 2 | 3 | 8 |
| Klein P | 3 | 2 | 2 | 7 |
| Caballero GC | 4 | 2 | 2 | 8 |
| Kruer RM | 4 | 2 | 3 | 9 |
| Bhullar IS | 4 | 2 | 2 | 8 |
| Gabriel WM | 3 | 2 | 3 | 8 |
| Javed G | 4 | 2 | 3 | 9 |
| Zangbar B | 3 | 2 | 2 | 7 |
| Khor D | 3 | 2 | 3 | 8 |
| Hazama A | 4 | 2 | 3 | 9 |
| Kwon SJ | 3 | 2 | 2 | 7 |
| Harris L | 3 | 2 | 3 | 8 |
| Nguyen JV | 4 | 2 | 3 | 9 |

A score of ≤5 indicates a high risk of bias
